# Retinitis Pigmentosa Reduces the Risk of Proliferative Diabetic Retinopathy: A Nationwide Population-Based Cohort Study

**DOI:** 10.1371/journal.pone.0045189

**Published:** 2012-09-28

**Authors:** Yuh-Fang Chen, Hsin-Yi Chen, Che-Chen Lin, Muh-Shy Chen, Pei-Chun Chen, I-Jong Wang

**Affiliations:** 1 Department of Ophthalmology, New Taipei City Hospital, New Taipei City, Taiwan; 2 Department of Ophthalmology, National Taiwan University Hospital, Taipei, Taiwan; 3 Graduate Institute of Clinical Medical Science, China Medical University, Taichung, Taiwan; 4 Department of Ophthalmology, China Medical University Hospital, Taichung, Taiwan; 5 Management Office for Health Data, China Medical University and Hospital, Taichung, Taiwan; 6 Graduate Institute of Epidemiology and Preventive Medicine, National Taiwan University College of Public Health, Taipei, Taiwan; Maastricht University Medical Center, The Netherlands

## Abstract

**Purpose:**

To study the association between retinitis pigmentosa (RP) and the progression of diabetic retinopathy (DR).

**Methods:**

Using the Longitudinal Health Insurance Database 2000 of Taiwan, we identified individuals with an initial diagnosis for RP during the period of 1997–2008. A non-RP comparison group, 10-fold frequency matched by sex, age, index year and the year of diabetes diagnosed, were randomly selected from the same database. The occurrence of DR was observed for all subjects until the end of 2009. The Kaplan-Meier curves were used to illustrate the cumulative probability of developing DR for the RP group and comparison groups. The hazard ratio (HR) of DR for the RP group relative to the comparison group was estimated using Cox proportional hazards model after adjusting for potential confounders.

**Results:**

The Kaplan-Meier curves were not statistically significant different between the RP group and the comparison group. However, the RP group had a higher cumulative probability of developing DR during the first six to seven years. The cumulative probability kept increasing and became higher in the comparison group but remained unchanged in the RP group. The HR for the RP patients comparing with the comparison group was 0.96 (95% confidence interval (CI) = 0.43–2.14). Stratified by severity, RP was associated with a non-statistically significant reduced risk of proliferative DR (PDR) (HR = 0.70, 95% CI = 0.16–3.14). The HR for non-proliferative DR (NPDR) was 1.08 (95% CI = 0.40–2.86).

**Conclusion:**

In this study, RP was not statistically significant associated with the incidence of DR.

## Introduction

Retinitis pigmentosa (RP) was first described by Donders in 1857 as an inflammatory disorder characterized by night blindness and pigmentary deposits in the retinae. [1] In 1861, Lirbreich determined this disorder to be hereditary rather than caused by inflammation and characterized PR as causing retinal “bone-spicule” pigmentary changes, a “waxy” pallor of the optic disc, and attenuated retinal vessels. [2] In 1872, Landolt found this condition was primarily due to photoreceptor degeneration and the vascular change was more likely the effect rather than the cause. [3] There has been increasing evidence to support Landolt’s opinion that vasoconstriction results from increased oxygen tension due to reduced oxygen consumption by the degenerated photoreceptors as well as retinal circulation closer to the choroid in the thinned retinae [4], [5], [6].

The incidence of diabetic retinopathy (DR) in patients with RP, in which the photoreceptor cells are apoptotic due to mutations of rhodopsin and other proteins, is very interesting from clinical and pathophysiological points of view. For instance, it is well known that the microvascular complications of diabetes seem more serious in the retina than in the brain due to a unique local factor in the retina, namely the photoreceptors. [7] In the retina, most oxygen consumption is utilized by photoreceptor rod cells because the abundant rods have the highest metabolic rate compared to any other cell types in our bodies, [8] especially during dark adaptation. [9] This process requires a great amount of oxygen and makes it easy for the retina to become hypoxic. Hypoxia induces vascular endothelial growth factor (VEGF), which increases vascular permeability, damages endothelial cells, and causes neovascularization [10].

Based on the severity of the retinal vascular changes, DR can be categorized into an earlier nonproliferative diabetic retinopathy (NPDR) stage and a more advanced proliferative diabetic retinopathy (PDR) stage. Vascular abnormalities in the former stage include capillary nonperfusion, dot hemorrhages, and microaneurysms, while the latter stage is characterized by neovascularization. [11] There are molecular biological explanations for the relationships between hyperglycemia and DR. First, the capillary endothelial cells in the retina are insulin-insensitive and so cannot regulate glucose well. [12], [13] Hyperglycemia hastens the production of reactive oxygen species (ROS), which leads to cell death. [14] Furthermore, advanced glycation end products may induce VEGF, [15] which promotes neovascularization. Finally, the capillary basement membrane thickens in response to hyperglycemia and causes tissue hypoxia, which also induces new vessel growth [16].

Using neonatal mice with classic inherited retinal degeneration (*Pdeb^rd1^/Pdeb^rd1^*) in a mouse model of oxygen-induced proliferative retinopathy, Lahdenranta et al. showed that reactive retinal neovascularization was abolished and VEGF failed to increase. They also presented a patient whose PDR regressed spontaneously while RP became more evident. Hence, they proposed that retinal neovascularization might stop progressing or even regress when the number of photoreceptors is reduced. [17] Similarly, using rhodopsin knockout mice (Rho^−/−^) as an RP model, de Gooyer et al. proved that hypoxia and VEGF-A increased and vascular density attenuated in the retina of wild-type diabetic mice, but these changes did not occur in Rho^−/−^ mice. [18] They concluded the loss of the outer retina might decrease oxygen consumption by the photoreceptors and thus prevent DR progression. This is the same reason retinal laser photocoagulation is helpful in controlling PDR, since it decreases photoreceptor bulk and hypoxia [19].

Based on the above evidence, we postulate that relative hyperoxia in RP could alleviate hypoxia in DR and thus slow down its progression to PDR. This assumption is confirmed by clinical experience showing that PDR is rarely comorbid with RP. Sternberg showed a negative correlation between the incidences of these two diseases. [20] Thus far, many studies have investigated the mechanisms of a complex interaction between RP and PDR using different animal models; [17], [18], [21], [22], [23], [24]however, long-term observation of a large number of patients is lacking in the literature. Hence, we designed a nationwide population-based cohort study with an observation time of more than 10 years to determine if the hypothesis can be validated clinically.

**Table 1:** The demographic characteristics and baseline comorbidities of the retinitis pigmentosa and comparison groups, 1997–2008.

	Comparison group	RP group	
Variable	n = 580 (%)	n = 58 (%)	p-value
Age, years			0.9591
Mean±SD*	55.4 (13.9)	55.3 (13.8)	
Sex			1.0000
Female	280 (48.3)	28 (48.3)	
Male	300 (51.7)	30 (51.7)	
Residential area			0.9621
Northern	261 (45.0)	28 (48.3)	
Central	113 (19.5)	11 (19.0)	
Southern	147 (25.3)	14 (24.1)	
Eastern	59 (10.2)	5 (8.6)	
Comorbidity			
Hypertension	335 (57.8)	33 (56.9)	0.8992
Glaucoma	35 (6.0)	11 (19.0)	0.0003
Cataract	82 (14.1)	38 (65.5)	<0.0001
Hyperlipidemia	310 (53.4)	27 (46.6)	0.3158
Heart disease	230 (39.7)	19 (32.8)	0.3046
Stroke	38 (6.6)	7 (12.1)	0.1177
PAD	45 (7.8)	4 (6.9)	0.8141

* t-test.

## Materials and Methods

### Database

The National Health Insurance Research Database (NHIRD) contains reimbursement claim data for the Taiwan National Health Insurance program, which covers more than 99% of the Taiwanese population. The database also contains demographic information, including sex, age, and area of residence. In order to ensure confidentiality, all personal identification numbers were encrypted before the databases were released to the public. For this study, we made use of part of the NHIRD, the Longitudinal Health Insurance Database 2000 (LHID2000). This includes historical claim data for one million subjects randomly selected from among people insured during 1996–2000. The International Classification of Diseases, Ninth Revision, Clinical Modification (ICD-9-CM) was used to code the outpatient and inpatient diagnoses.

**Table 2:** Incidence of diabetic retinopathy and the hazard ratio for the retinitis pigmentosa and comparison groups.

	Comparison group			RP			Hazard ratio (95% CI)	
	Event	PY	Incidence†	Event	PY	Incidence†	Crude	Adjusted‡
Total	80	2890	27.68	8	281	28.49	1.04(0.50–2.15)	0.96(0.43–2.14)
NPDR	49	2890	16.96	6	281	21.37	1.28(0.55–2.98)	1.08(0.40–2.86)
PDR	31	2890	10.73	2	281	7.12	0.66(0.16–2.77)	0.70(0.16–3.14)

PY: person-years; CI: confidence interval; NPDR, nonproliferative diabetic retinopathy; PDR, proliferative diabetic retinopathy.

† per 1000 person-years.

‡ adjusted for sex, age, residence area, hypertension, glaucoma, cataracts, and heart disease.

### Study Subjects

This was a nationwide population-based cohort study. Using outpatient or inpatient claims, we identified individuals who had an initial diagnosis of RP (ICD-9-CM code 362.74) and had been diagnosed with diabetes (ICD-9-DM 250 and A-code A181) during the period 1997–2008. An index date for a RP patient was his (or her) date of diagnosis. Subjects in the comparison group, 10-fold frequency matched by sex, age, index year and the year of diabetes diagnosis, were randomly selected from among individuals who had been diagnosed with diabetes but not been diagnosed with RP. A patient was defined as having developed the event, DR, if the individual had a subsequent physician visit for ICD-9-CM code 362.0x. The events were further classified by the severity of the disease. Patients were defined as having PDR if they had a diagnosis coded ICD-9-CM 362.02 or if they had undergone any surgery for DR (including laser eye surgery or vitreous surgery); all others were classified as having NPDR. Patients who had been diagnosed with DR before the index date were excluded from this study.

For subjects whose diabetes diagnosis occurred before index date, the follow-up person-years were calculated starting from the index date; for those whose diabetes was diagnosed after index date, the follow-up began at the date of diabetes diagnosis. The follow-up terminated when the patients withdrew from the insurance program, when DR occurred or at December 31, 2009.

We also searched the database to see if comorbidities of DR were present before the date of follow-up started, including hypertension (ICD-9-CM 401–405 and A code A260 and A269), glaucoma (ICD-9-CM 365), cataracts (ICD-9-CM 366.1, 366.2, 366.3, 366.5 and 366.9), hyperlipidemia (ICD-9-CM 272 and A code A182), heart disease (ICD-9-CM 410–429 and A code A270, A279–A281 and A289), stroke (ICD-9-CM 430–438) and peripheral arterial disease (PAD, ICD-9-CM 440–448 [except for 440.0 and 440.1]).

**Table 3:** Incidence of diabetic retinopathy and risk for the retinitis pigmentosa and comparison groups with different comorbidities.

	Comparison group			RP			Hazard ratio (95% CI)		
	Event	PY	Incidence†	Event	PY	Incidence†	Crude	Adjusted‡	p
Hypertension									0.5032
No	24	1270	18.89	3	123	24.47	1.33(0.40–4.41)	1.98(0.49–8.04)	
Yes	56	1619	34.58	5	158	31.6	0.92(0.37–2.29)	0.77(0.29–2.10)	
Glaucoma									0.6250
No	74	2719	27.22	5	232	21.6	0.80(0.32–1.99)	0.72(0.27–1.88)	
Yes	6	171	35.12	3	49	60.84	1.73(0.43–6.94)	–	
Cataract									0.9821
No	73	2564	28.47	0	105	0	–	–	
Yes	7	326	21.5	8	176	45.54	2.21(0.80–6.11)	1.98(0.60–6.56)	
Hyperlipidemia									0.5345
No	36	1358	26.5	5	137	36.48	1.40(0.55–3.57)	1.43(0.52–3.93)	
Yes	44	1532	28.73	3	144	20.87	0.73(0.23–2.34)	0.57(0.15–2.12)	
Heart disease									0.9762
No	51	1753	29.09	8	185	43.24	1.48(0.70–3.13)	1.43(0.60–3.39)	
Yes	29	1137	25.51	0	96	0	–	–	
Stroke									0.9820
No	75	2737	27.4	8	257	31.1	1.14(0.55–2.37)	1.03(0.46–2.32)	
Yes	5	153	32.73	0	24	0	–	–	
PAD									0.4826
No	69	2720	25.37	7	266	26.27	1.05(0.48–2.28)	1.05(0.44–2.51)	
Yes	11	170	64.69	1	14	69.51	1.26(0.16–9.91)	0.07(0.002–2.14)	

PY: person-years; CI: confidence interval.

† per 1000 person-years.

‡ adjusted for sex, age, residence area, hypertension, glaucoma, cataracts, and heart disease.

p value for interaction.

### Statistical Analysis

We compared the distribution of demographic factors and comorbidities between the RP and comparison groups using the chi-square test for categorical variable and t-test for continuous variable. The incidence density of DR was calculated by using the number of incident DR dividing by person-years at risk in both groups. The Kaplan-Meier survival curves were used to illustrate the cumulative probability of developing DR among RP patients and subjects in the comparison groups. The difference between the two curves was examined by using the log-rank test. The hazard ratio (HR) (and 95% confidence interval [CI]) of DR for the RP group comparing with the comparison group was estimated using Cox proportional hazards models after adjusting for potential confounders. We examined the proportional hazard assumption by adding an interaction term between time and the variable RP. No significant interaction was observed when time was defined as a continuous variable (p = 0.0641) or a categorical variable with year 5 as the cutoff point (p = 0.3241). Statistical analysis and data management were undertaken using SAS 9.1 software (SAS Institute, Cary, NC) and the survival curve was drawn using R software. The level of statistical significance was set at a p value less than 0.05.

**Figure 1:**
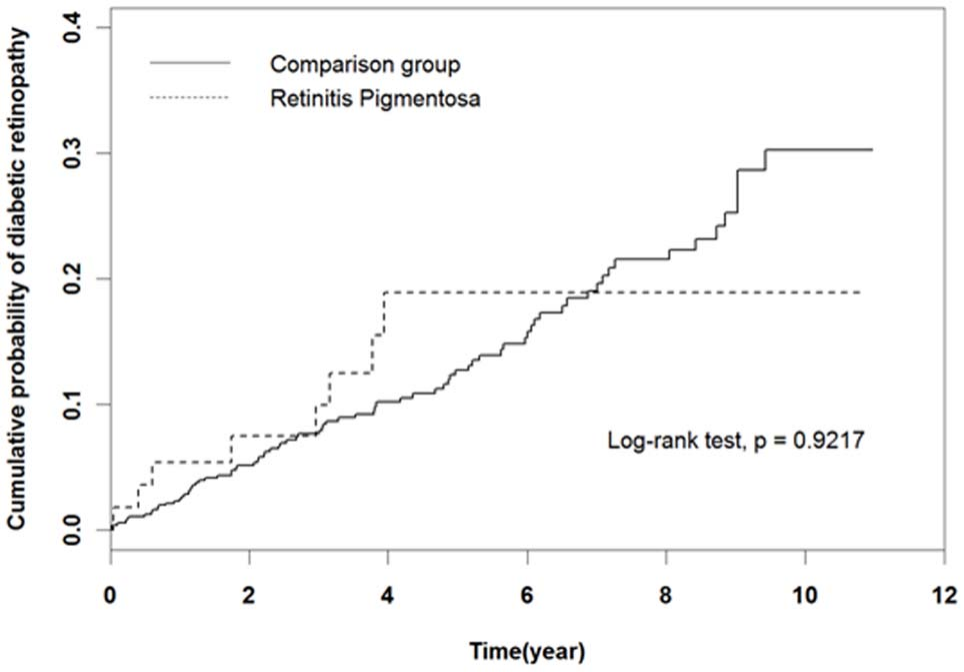
Kaplan-Meier methods estimating cumulative probability of developing diabetic retinopathy among patients with retinitis pigmentosa and subjects in the comparison group.

## Results

This study included 58 RP patients and 580 comparison individuals identified during 1997–2008. These two groups had a very similar distribution of the matching variables age and sex (Table 1). No significant difference in residential area was observed between the two groups. Compared with the comparison group, RP patients were much more likely to have glaucoma (19.0% vs. 6.0%) and cataracts (65.5% vs. 14.1%) (p value <0.05 for both). The RP patients had a lower prevalence of hyperlipidemia and heart disease and a higher prevalence of stroke, but the difference was not statistically significant. The prevalence of hypertension and PAD was similar between the two groups.

Figure 1 shows the Kaplan-Meier curve estimating cumulative probability of developing DR over a median follow up of 4.2 years for the comparison group and 4.0 years for the RP group. Overall, the survival curves were not significantly different between the RP and comparison groups (log-rank test = 0.921). During the first six to seven years of follow-up, the cumulative probability of DR was higher in the RP group than in the comparison group. After that time, however, the cumulative probability in the comparison group was higher. The last event of the RP group occurred at the 3.9 year of follow up.

During the follow-up period, the incidence density of DR per 1000 person-years was 28.49 in the RP group (8 events) and 27.68 (80 events) in the comparison group; the HR, adjusted for age, sex, residential area, glaucoma, cataracts, heart disease, and hypertension, was 0.96 (95% CI = 0.43–2.14) (Table 2). Further analyses revealed possible differences in risk by severity of DR, but the HRs were not statistically significant. Relative to the comparison group, the HR for the RP patients was 1.08 (95% CI = 0.40–2.86) for developing NPDR and 0.70 (95% CI = 0.16–3.14) for PDR.

Table 3 displays the incidence and HR of DR in the RP and comparison groups stratified by comorbidities. RP tended to be associated with an increased risk of developing DR in individuals with cataract and in individuals without the comorbidity, including hypertension, hyperlipidemia and heart disease. In contrast, a possibly reduced risk for the RP group compared to the comparison group was observed in subjects without glaucoma and in individuals with hypertension and hyperlipidemia. However, all HRs in the stratified analyses were not statistically significant. No significant interactions between these comorbid disorders and RP were observed.

## Discussion

In our study, it is interesting to note the estimated cumulative probability of developing DR in the RP group was higher than that in the comparison group during the first six to seven years. Thereafter, the cumulative probability in the comparison group kept increasing but remained stable in the RP group. On the other hand, we also noticed the HRs might differ for different stages of DR; however, they are not statistically significant (the HR for the RP patients was 1.09 to develop NPDR and 0.67 to develop PDR). Both the above findings could be explained by the interaction of the pathophysiological processes of DR and RP. Although PDR is characterized by increased vasopermeability and neovascularization, there was evidence DR starts with vasoregression. [25] In the very early stage, angiopoietin-2 is known to be upregulated in response to hyperglycemia to induce and exaggerate pericyte dropout. High glucose levels cause ROS overproduction, which also damages endothelial cells and leads to further vasoregression. [26] After hypoxia becomes evident as a result of vasoregression, VEGF and angiopoietin-2 cooperate to induce endothelial cell proliferation, pericyte activation, and finally neovascularization [27], the PDR stage. According to these findings, RP and the early stage of DR (the NPDR stage) have a similar pathophysiological process to vasoregression so that the severity of DR might be enhanced in the presence of RP. The mechanism of vasoregression is hyperoxia due to photoreceptor apoptosis in RP and hyperglycemia in NPDR. Later, the pathogenesis of PDR turns into neovascularization, which is opposite to that of RP. As a consequence, comorbid RP decreases oxygen demand, improves hypoxia, and slows down the progression of PDR.

In moderate and advanced RP, vascular attenuation is very common in addition to the wax-pale disc and the bone-spicule-like pigment changes. This phenomenon is opposite to PDR, in which neovascularization is prominent. Grunwald et al. hypothesized that vascular attenuation in RP was due to vasoconstriction and reduced retinal blood flow in response to the loss of the photoreceptors and decreased oxygen consumption. [28] Penn et al. established a transgenic mouse model of autosomal dominant RP and examined the role of hyperoxia resulting from photoreceptor apoptosis in vascular atrophy. They proved ambient hypoxia not only stopped capillary degeneration, but also stimulated capillary growth in the deep and superficial vascular beds. [23] More recently, Padnick-Silver used Abyssinian cats with hereditary retinal degeneration to study the role of retinal oxygen in vascular attenuation and photoreceptor degeneration. They found the loss of photoreceptors lowered the oxygen demand, led to hyperoxia, and caused retinal vascular atrophy. [24] Salom et al. collected aqueous humor samples from patients with RP and found the concentration of VEGF-A was significantly decreased in patients with RP compared to control subjects. [22] They proposed that the loss of RPE cells, which are important sources of VEGF, and retinal hyperoxia due to photoreceptor degeneration together cause VEGF-A reduction in RP. In addition to the effects on endothelial mitosis and vascular permeability and since VEGF-A also has neurotrophic and neuroprotective effects on neuronal growth, differentiation, and survival, [29], [30] the authors postulated that the lack of VEGF-A might lead to retinal vascular narrowing, fibrotic change, and the loss of neuroprotection in patients with RP.

A major strength of this study is our use of a large dataset randomly sampled from the claims data of National Health Insurance program, which covers nearly the whole Taiwanese population. [31], [32] Hence, we could collect a representative sample of Taiwanese patients with RP which leaves little room for selection bias. The large pool of potential comparison subjects allows us to select a well-matched comparison group in terms of age, sex and date of diabetes diagnosis. However, even though the database is large, containing one million subjects, there were only 58 RP patients with diabetes, and eight of them had DR. This small number of patients with DR might explain the insignificant statistical results in our analyses.

In conclusion, we provided a well-controlled nationwide population-based cohort study over a long period of observation time. RP was not significantly associated with the risk of developing DR. Further study on this issue may help clarify whether the association between RP and DR differs over time and confirm the possibly effect of RP on the reduction in risk of severe DR known as PDR.
